# Impact of signal-to-noise ratio and contrast definition on the sensitivity assessment and benchmarking of fluorescence molecular imaging systems

**DOI:** 10.1117/1.JBO.30.S1.S13703

**Published:** 2024-07-18

**Authors:** Elena Kriukova, Ethan LaRochelle, T. Joshua Pfefer, Udayakumar Kanniyappan, Sylvain Gioux, Brian Pogue, Vasilis Ntziachristos, Dimitris Gorpas

**Affiliations:** a Institute of Biological and Medical Imaging, Helmholtz Zentrum München, Neuherberg, Germany; b Technical University of Munich, School of Medicine and Health, Chair of Biological Imaging at the Central Institute for Translational Cancer Research (TranslaTUM), Munich, Germany; c QUEL Imaging, White River Junction, Vermont, United States; d Thayer School of Engineering at Dartmouth College, Hanover, New Hampshire, United States; e Center for Devices and Radiological Health, Food and Drug Administration, Silver Spring, Maryland, United States; f Intuitive Surgical, Aubonne, Switzerland; g University of Strasbourg, ICube Laboratory, Strasbourg, France; h University of Wisconsin Madison, Department of Medical Physics, Madison, Wisconsin, United States; i Technical University of Munich, Munich Institute of Robotics and Machine Intelligence (MIRMI), Munich, Germany

**Keywords:** fluorescence molecular imaging, composite phantom, standardization, benchmarking, image analysis, signal-to-noise ratio, contrast

## Abstract

**Significance:**

Standardization of fluorescence molecular imaging (FMI) is critical for ensuring quality control in guiding surgical procedures. To accurately evaluate system performance, two metrics, the signal-to-noise ratio (SNR) and contrast, are widely employed. However, there is currently no consensus on how these metrics can be computed.

**Aim:**

We aim to examine the impact of SNR and contrast definitions on the performance assessment of FMI systems.

**Approach:**

We quantified the SNR and contrast of six near-infrared FMI systems by imaging a multi-parametric phantom. Based on approaches commonly used in the literature, we quantified seven SNRs and four contrast values considering different background regions and/or formulas. Then, we calculated benchmarking (BM) scores and respective rank values for each system.

**Results:**

We show that the performance assessment of an FMI system changes depending on the background locations and the applied quantification method. For a single system, the different metrics can vary up to ∼35  dB (SNR), ∼8.65  a.u. (contrast), and ∼0.67  a.u. (BM score).

**Conclusions:**

The definition of precise guidelines for FMI performance assessment is imperative to ensure successful clinical translation of the technology. Such guidelines can also enable quality control for the already clinically approved indocyanine green-based fluorescence image-guided surgery.

## 1 Introduction

Fluorescence molecular imaging (FMI) has made great advances in clinical translation over the last few years.^1^ Driven by these advances, technologies at the forefront of the field are evolving rapidly, particularly in the areas of device design, fluorescent agents, image processing algorithms, and performance assessment metrics.^2^ Consequently, the number of imaging devices and their applications is increasing.

Moreover, following the first-in-human application of FMI in 2011 by van Dam et al.,^3^ numerous clinical studies have been completed or are currently ongoing. A major outcome of all this activity is the recent approvals by the US Food and Drug Administration (FDA) of ∼20 fluorescence-guided clinical imaging systems^4^ as well as 3 tracers for surgical guidance: (1) 5-aminolevulinic acid (5-ALA/Gleolan®; Photonamic GmbH and Co., KG, Pinneberg, Germany) for use as an intra-operative optical imaging agent in patients with suspected high-grade gliomas,^5^ (2) hexaminolevulinate (HAL, available as Hexvix, Photocure ASA, Oslo, Norway, and Cysview Photocure Inc., Princeton, New Jersey, United States) for use in non-muscle-invasive bladder cancer,^6^ and (3) pafolacianine (Cytalux, On Target Laboratories LLC, West Lafayette, Indiana, United States) for intraoperative imaging of folate receptor-positive ovarian and lung cancers.^7^^,^^8^ All this activity has highlighted the need for better and user-independent standardization procedures that would allow for system characterization, performance monitoring, data referencing, and comparison, even among markedly different systems. This is, also, very relevant to the fluorescent image-guided surgery (FIGS), given the FDA clearance of multiple FIGS devices for imaging with indocyanine green (ICG) and other contrast agents.^9^ Addressing this need is essential for ensuring optimal impact and wider clinical acceptance of FMI and FIGS. ^10^

Over the past few years, numerous studies on phantom development and standardization procedures, as well as attempts to achieve consensus in the community, have been reported.^1^^,^^2^^,^^9^^,^^11^^–^^19^ Thus far, methods and reference targets for system evaluation and comparison have been developed on an individual basis, but a universal cross-platform metric for image fidelity evaluation has yet to be developed.^16^

Currently, the sensitivity of FMI systems is assessed mostly using the signal-to-noise ratio (SNR) and/or contrast metrics.^2^^,^^12^^,^^17^^,^^20^ It has been shown, however, that the definition of the background can play a significant role in the interpretation of the acquired images, especially during tissue imaging.^21^^–^^24^ For example, Chen et al.^22^ and Hoogstins et al.^21^ reported that background estimation significantly affected quantification results for bulk-stained tissue fluorescence imaging and intraoperative/*ex vivo* fluorescence imaging, respectively, using metrics including SNR, signal-to-background ratio (SBR), and contrast-to-noise ratio (CNR). Widen et al.^23^ demonstrated the impact of region of interest (ROI) sizes on overall signal and mean fluorescence intensity by analyzing fluorescent probes in animal experiments. Dijkhuis et al.^24^ also demonstrated the effect of manually selected ROI in fluorescent data analysis and proposed semi-automatic methods for objective assessment of fluorescent signals in resected tissue. In view of the theoretical effect described above, Azargoshasb et al.^25^ quantified how fluorescent SBR influences the robotic surgical performance of participants (n=16) during an exercise with a custom grid phantom. On the other hand, Palma-Chavez et al.^26^ reported 15 different SNRs and five contrast formulas that are currently used in the field of optoacoustics, indicating that the lack of consensus is not only limited to FMI applications. The plethora of background definitions, as well as the different quantification formulas used across multiple studies, emphasize the importance of reaching a wide consensus for performance assessment and quality control of FMI systems.

Indeed, despite the fact that SNR and contrast are the most commonly used metrics for the sensitivity assessment of various systems,^1^^,^^26^ there are only a few studies comparing different FIGS systems, most of which are optimized for ICG imaging.^9^^,^^19^^,^^27^ In addition, the formulas used to calculate SNR and contrast and methods for evaluating background ROIs vary across different studies. In a recent study, LaRochelle et al.^28^ demonstrated the influence of background definition in SBR, SNR, CNR, and contrast-to-variability ratio through measurements on anthropomorphic three-dimensional (3D)-printed phantoms. However, to the best of our knowledge, there is no study quantifying the effect of the combined variation (ROIs and metrics formulas) on performance assessment. An in-depth testing and evaluation of current strategies are crucial to raise community awareness of existing limitations, to spur effective development of the technology, and to set the performance limits that are required for regulatory approvals.

Building on the assumption that the SNR and contrast metrics depend on the selection of background ROIs and quantification formulas, herein, for the first time, we systematically investigate and showcase this dependence with regard to the sensitivity assessment of markedly different FMI systems.

In specifics, using six near-infrared FMI systems, we captured fluorescence images of a composite rigid phantom previously developed by our group.^11^^,^^18^^,^^19^ We then assessed the sensitivity^19^ of those systems using six previously published formulas for SNR and contrast^17^^,^^29^^–^^33^ and two background locations. Moreover, based on these metrics, we quantified the corresponding benchmarking (BM) scores,^19^ and the systems were ranked based on these scores.

Recently, we called attention to the need for a commonly accepted phantom to promote good imaging practices during the development of FMI systems or their use in clinics.^1^ We now pinpoint additional needs to consistently define ROIs and use common quantification formulas for SNR and contrast. Answering these needs will enable consistency, allow data comparison and referencing, and advance the quality and performance of FMI systems. These improvements will promote wide acceptance and usage of FMI as a tool for interventional and endoscopic procedures.

## 2 Materials and Methods

### 2.1 FMI**Systems**

**Fig. 1:**
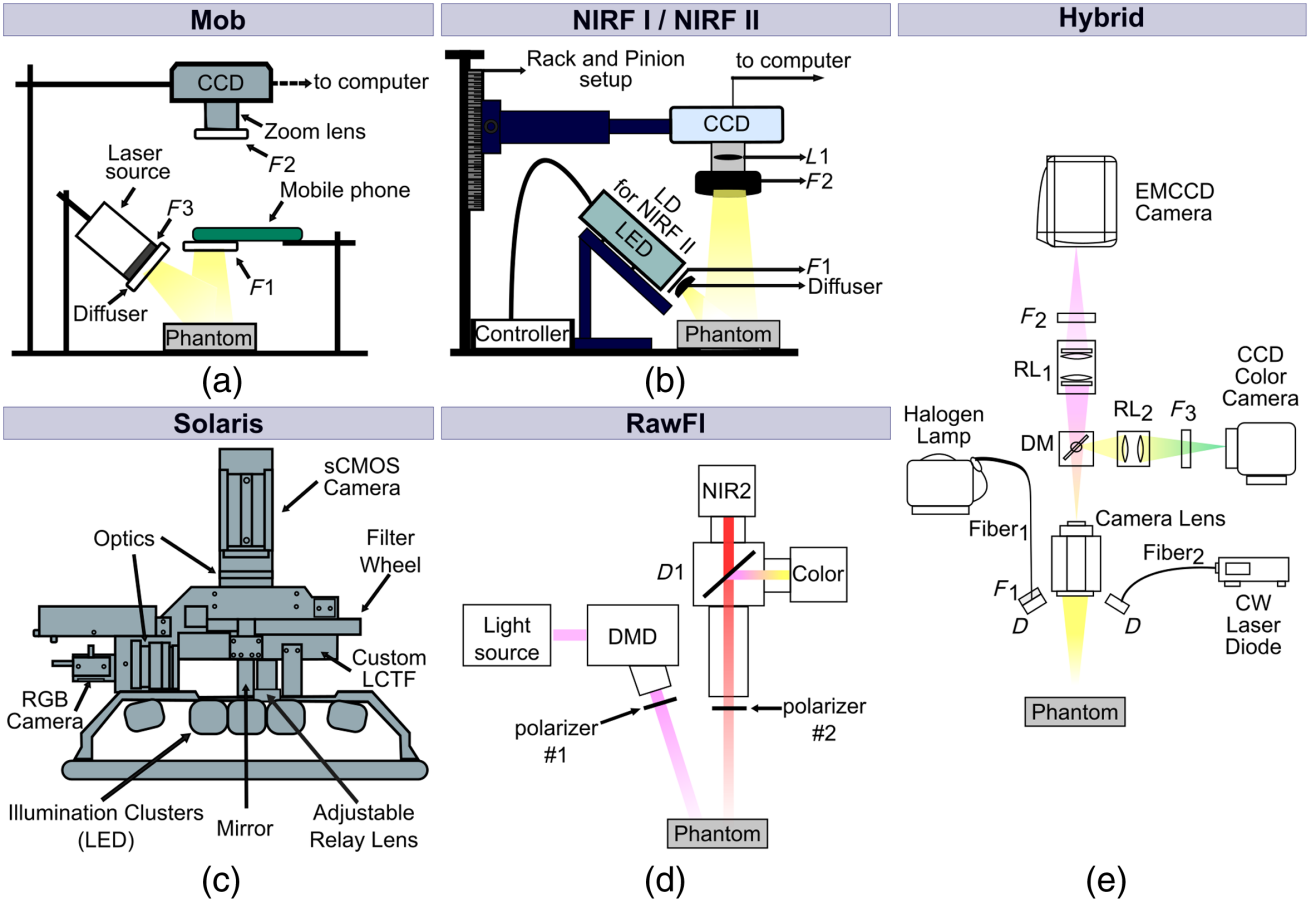
Schematic diagrams of the fluorescence imaging systems used in the study. (a) Mob—adapted with permission from Ghassemi et al.^34^ (b) NIRF I/NIRF II—adapted from Kanniyappan et al.^35^ (c) Solaris—adapted and modified from Behrooz et al.^38^ (d) RawFl—adapted from Ségaud et al.^36^ (e) Hybrid—adapted with permission from Glatz et al.^37^ CCD, charge-coupled device; F, filter; LED, light-emitting diode; LD, laser diode; L, lens; EMCCD, electron-multiplying charge-coupled device; RL, relay lens; DM, dichroic mirror; CW, continuous wave; sCMOS, scientific complementary metal oxide semiconductor; LCTF, liquid crystal tunable filter; D, diffuser; DMD, digital micro-mirror device; NIR2, near-infrared camera.

For this study, we used six fluorescence imaging systems distributed in different labs in the United States and Europe. The main specifications of each system, as well as the adopted phantom imaging protocols, are summarized in Table 1, while the corresponding system schematics are presented in Fig. 1. All measurements were conducted in darkness to eliminate the influence of ambient light on the results.

**Table 1:** FMI systems used in this study and the corresponding imaging protocols.

Name	Sensor	Bit depth (bit)	Resolution (pixels)	Wavelengths (nm, excitation/emission)	Exposure time (sec)	Working distance (mm)	Fluence rate^a^ ($\mathrm{mW}/{\mathrm{cm}}^{2}$)	Reference
Mob	CMOS camera	8	3264×2448	785/825	0.1	150	12.5	34
NIRF I	Apogee Camera-Alta U2020ML	16	1600×1200	785/825	1	750	0.74	35
NIRF II	Kodak KAI-2020M	16	2758×2208	785/825	1	750	12.5	Not available
Solaris	Fluorescence sCMOS camera	16	2200×2500	730/800	dependent on the video rate	700	10	PerkinElmer®Solaris
RawFl	sCMOS camera	16	1024×1024	760/800	0.2	450	1.5	36
Hybrid	EMCCD camera	16	512×512	750/810-90	0.1	150	15.5	37

a At the phantom surface.

Mob is a mobile phone-based near-infrared fluorescence (NIRF) imaging system previously,^34^ where its spectral sensitivity was documented. It involves a 1W 785-nm laser diode, an 800-nm short-pass excitation filter (84-729, Edmund Optics, Barrington, New Jersey, United States), and a long-pass emission filter with a cutoff wavelength at 825 nm (86-078, Edmund Optics) for the detection. The phone camera is based on an 8-bit complementary metal oxide semiconductor (CMOS) sensor with an f/2.4 aperture lens (Eigen Imaging, Inc., San Diego, California, United States) and a near-infrared blocking filter, which was removed during this study.

NIRF I is a custom benchtop NIRF imaging system^35^ with a light-emitting diode (M780L3, Thorlabs, Inc., Newton, New Jersey, United States) centered at 780 nm and power of 200 mW. The same optical filters used with the Mob system were also used in the NIRF I imaging system. A 16-bit charge-coupled device camera (Alta U2000, Apogee Imaging Systems, Roseville, California, United States) coupled with a zoom lens (7-mm focal length, f/3.9, Tamron, Commack, New York, United States) was used for the detection of the emitted fluorescence.

NIRF II is an updated version of the NIRF I imaging system. Its main improvement is the replacement of the imaging sensor with the more sensitive Kodak KAI-2020M (Image Sensor Solutions Eastman Kodak Company, Rochester, New York, United States), while fluorescence was induced by a laser diode at 785 nm and 1W power, instead of the light-emitting diode present in NIRF I system.

Solaris is an open-air commercially available fluorescence imaging system by PerkinElmer (Waltham, Massachusetts, United States). The Solaris system is designed for research applications, including preclinical studies for advanced molecular-guided surgery, and drug efficacy and safety measurements.

RawFl is a custom-built setup^36^ with a filtered 760-nm laser diode (LDX Optronics, Maryville, Tennessee, United States) light source, a 16-bit scientific complementary metal oxide semiconductor (sCMOS) camera (pco.edge 5.5, PCO AG, Kelheim, Germany) as a detector and polarizers (PPL05C; Moxtek, Orem, Utah, United States) for minimizing the contribution from specular reflections at the surface of the sample.

Hybrid is a custom-built system combining fluorescence and color imaging and has been described previously.^37^ Fluorescence excitation is achieved using a laser diode (FLX-750-1500 M-100-9 MM Frankfurt Laser Company, Friedrichsdorf, Germany) and detection with an electron-multiplying charge-coupled device (EMCCD, DV897DCS-BV, Andor Technology, Belfast, United Kingdom).

### 2.2 Standardization Phantom

**Fig. 2:**
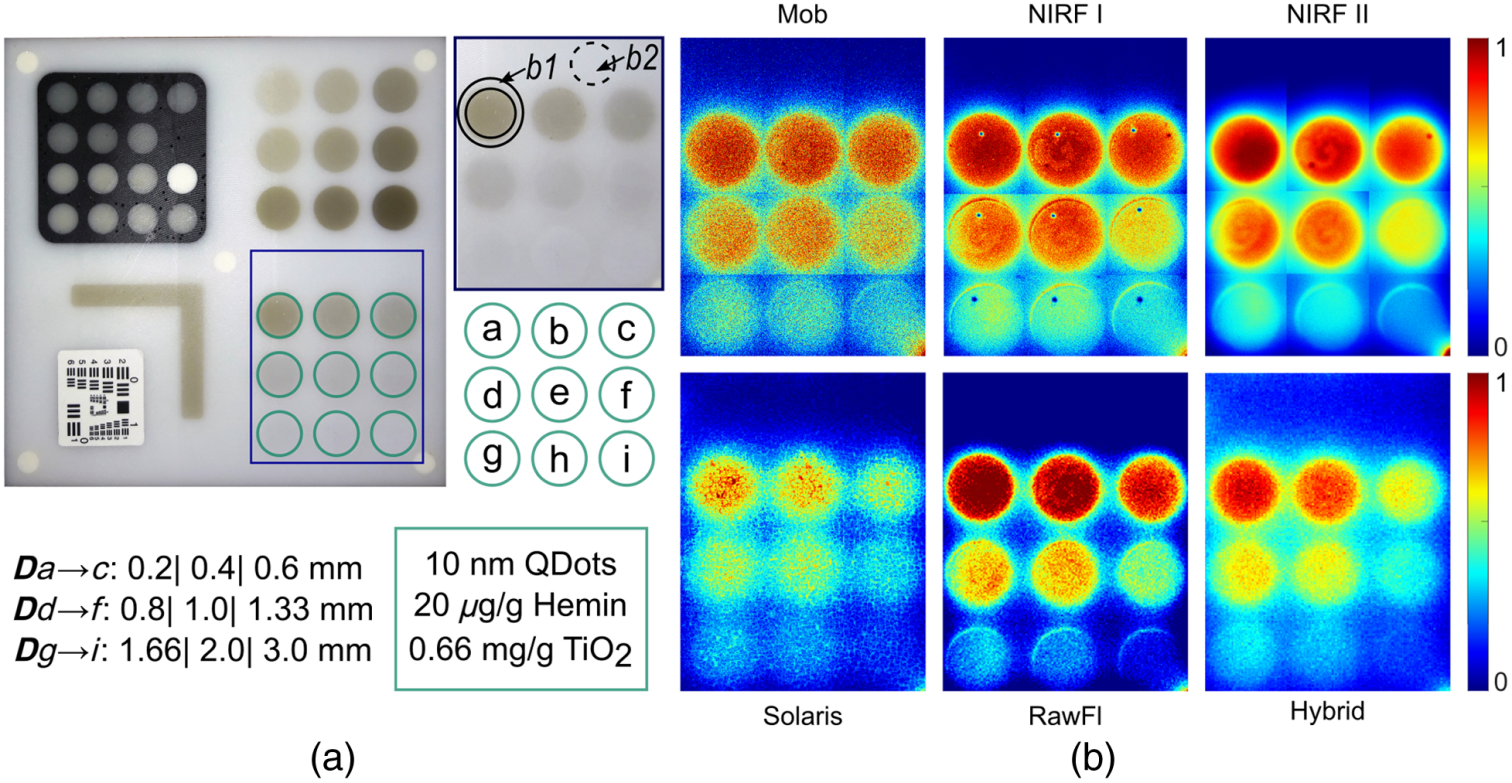
Sensitivity versus depth phantom region. (a) An illustration of the composite phantom used in this study, with the sensitivity versus depth wells highlighted and enlarged. Arrows denote two areas (b1 and b2) used as background regions. The depth of the phantom wells (bottom left, Dx where x=a,b,c…) indicates the distance from the top surface of the phantom to each fluorescent inclusion. The concentrations of different constituents are the same for all inclusions. Qdots, quantum dots for fluorescence; Hemin, bovine hemin; and ${\mathrm{TiO}}_{2}$, nanoparticles (see Sec. 2.1). (b) Fluorescence images normalized to their corresponding maxima as acquired by the six systems employed in the study (see Table 1 for the description of each system).

The composite phantom shown in Fig. 2(a)^19^ was used to quantify the SNR and contrast from images acquired by the six systems. The application of the phantom as a fluorescence standard for performance assessment, quality control, and comparison of markedly different systems through a single image has been described in detail in previous studies.^11^^,^^18^^,^^19^ In the current study, however, the SNR and contrast were evaluated only on the “sensitivity versus depth” region of the phantom [see Fig. 2(a)]. This region includes (1) the transparent polyurethane (WC-783 A/B, BJB Enterprises, Tustin, California, United States) matrix base, with 0.00875 mg/g alcohol-soluble nigrosin (Sigma Aldrich, St. Louis, Missouri, United States) and 1.5 mg/g ${\mathrm{TiO}}_{2}$ nanoparticles (titanium IV oxide; Sigma Aldrich) for mimicking absorption and scattering, and (2) nine equally sized circular wells, made of the same polyurethane base with 20  μg/g bovine hemin (≥90% pure; Sigma Aldrich) and 0.66 mg/g ${\mathrm{TiO}}_{2}$ for absorption and scattering and 10-nM organic quantum dots (Qdot 800 ITK, Thermofisher Scientific, Waltham, Massachusetts, United States) for fluorescence. As shown in Fig. 2(a), the nine wells were embedded into the phantom matrix at distances of 0.2, 0.4, 0.6, 0.8, 1.0, 1.33, 1.66, 2.0, and 3.0 mm, respectively, from the phantom’s top surface.

### 2.3 Data Processing

The sensitivity versus depth phantom region [Fig. 2(a)] was extracted from the fluorescence images acquired by each system, and the SNR and contrast metrics were quantified by adopting the formulas in Table 2.

**Table 2:** Formulas for calculating SNR and contrast.

Name		Formula	Description	Reference
${\mathrm{SNR}}_{1}$		$\mathrm{SNR}=\frac{n}{\sigma }=\sqrt{n}$	n—number of photons on the detector; σ—the noise associated with the detector (i.e., standard deviation)	29
		$\sigma =\sqrt{n}$		
${\mathrm{SNR}}_{2}^{b1}$		$\mathrm{SNR}=\frac{S}{\sqrt{S+N}}$	S—mean foreground signal pixel intensity; N—mean background noise pixel intensity	30
${\mathrm{SNR}}_{2}^{b2}$				
${\mathrm{SNR}}_{3}^{b1}$		$\mathrm{SNR}=\frac{\mu_{S-N}}{\sigma_{S}}\;$	$\mu_{S-N}$—mean signal after background subtraction; $\sigma_{S}$—standard deviation of the signal	31
${\mathrm{SNR}}_{3}^{b2}$				
${\mathrm{SNR}}_{4}^{b1}$		$\mathrm{SNR}=\frac{S-N}{\sigma_{N}}$	S—mean signal pixel intensity; N—mean background noise pixel intensity, $\sigma_{N}$—background standard deviation	17
${\mathrm{SNR}}_{4}^{b2}$				
Michelson contrast	$C_{M}^{b1}$	$C_{M}=\frac{I_{\max}-I_{\min}}{I_{\max}+I_{\min}}$	$I_{\max},\;I_{\min}$—maximum pixel intensity and minimum background pixel intensity, respectively	32
	$C_{M}^{b2}$			
Weber contrast	$C_{W}^{b1}$	$C_{W}=\frac{I_{s}-I_{b}}{I_{b}}$	$I_{s},I_{b}$—maximum foreground and minimum background light intensity, respectively	33
	$C_{W}^{b2}$			

First, all images of the phantom wells from the region sensitivity versus depth were converted into binary images using the MATLAB function “imbinarize,” with the default option of thresholding using the Otsu method (MathWorks, Natick, Massachusetts, United States), and the location and radius of each well were obtained using the “imfindcircles” function. The extracted wells were then adjusted to match the size and location of the phantom wells based on the phantom design template, which ensured all wells preserved the same size within an image, regardless of the per-well fluorescence intensity distribution. Using this information, one mask was created to extract the average fluorescence intensity and standard deviation values from each well. A second mask, consisting of (i) the annuli between each well and concentric to the wells’ circles with a 40% larger radius (termed ROI b1) and (ii) a well-sized circular area in the non-fluorescent region of the phantom (termed ROI b2), was also created to quantify the average intensity and corresponding standard deviation values from the background ROIs (Fig. 2). The ROI b1 is adjacent to the wells that produce fluorescence signal, where fluorescence leakage to the neighboring phantom areas influences the ROI’s intensity values. This is frequently adopted as a strategy for background definition in multiple studies.^9^^,^^28^ The second ROI, b2, is located far from fluorescent wells and thus is not affected by fluorescence leakage. This is another frequently adopted definition of background, especially for studies where autofluorescence or diffusion is strong in the proximity of the target.^13^

To investigate the impact of chosen ROIs and quantification formulas (Table 2) on the BM of FMI systems, we calculated BM scores for each system as derived from the sensitivity versus depth phantom region using the method previously described.^19^ Briefly, the BM scores were defined as

BM=sMAPE/N, (1)

where sMAPE is the symmetric mean absolute percentage error of the SNR and contrast metrics that have been quantified for the various formulas of Table 2 and for the two background regions shown in Fig. 2(a). The sMAPE is calculated as

$$\mathrm{sMAPE}=\;\frac{1}{n}\cdot \overset{n}{\underset{i=1}{\sum }}\frac{\left|X_{i}-Y_{i}\right|}{\left|X_{i}|+|Y_{i}\right|},$$ (2)

where nis the number of phantom wells included in the metrics’ evaluation (n=9), $X_{i}$ is the value of the metric result (i.e., SNR or contrast), and $Y_{i}$ is the reference value. For the BM score quantification, we considered normal signal distributions, according to which a measurement is assumed to present 95% confidence if the signal is twofold the noise level. This results in reference values of 6 dB for SNR, 0.33 for Michelson contrast, and 1 for Weber contrast.^19^

Since the scope of this work is to assess how the SNR and contrast change depending on the application of different formulas and/or ROIs, all data processing was implemented on single images of the phantom acquired by the six systems. The repeatability and error analysis of the quantification of those two metrics have been recently reported by our group elsewhere.^39^

## 3 Results

Employing the six FMI systems described in Table 1, we imaged the composite phantom of Fig. 2(a) and isolated the sensitivity versus depth region from the acquired images, as shown in Fig. 2(b). As expected, the markedly different systems yield subjectively different images from the same area of the same phantom, which highlights the importance and need for FMI standardization to ensure a consistently high degree of performance and facilitate clinical translation.

The ROIs used for the quantification of SNR and contrast are shown in the top-right inset of Fig. 2(a). The arrows point out the two areas (b1 and b2) used for background calculation. These locations were chosen based on different studies assessing the performance of FMI systems^13^^,^^28^ and according to phantom constituents and geometry.

**Fig. 3:**
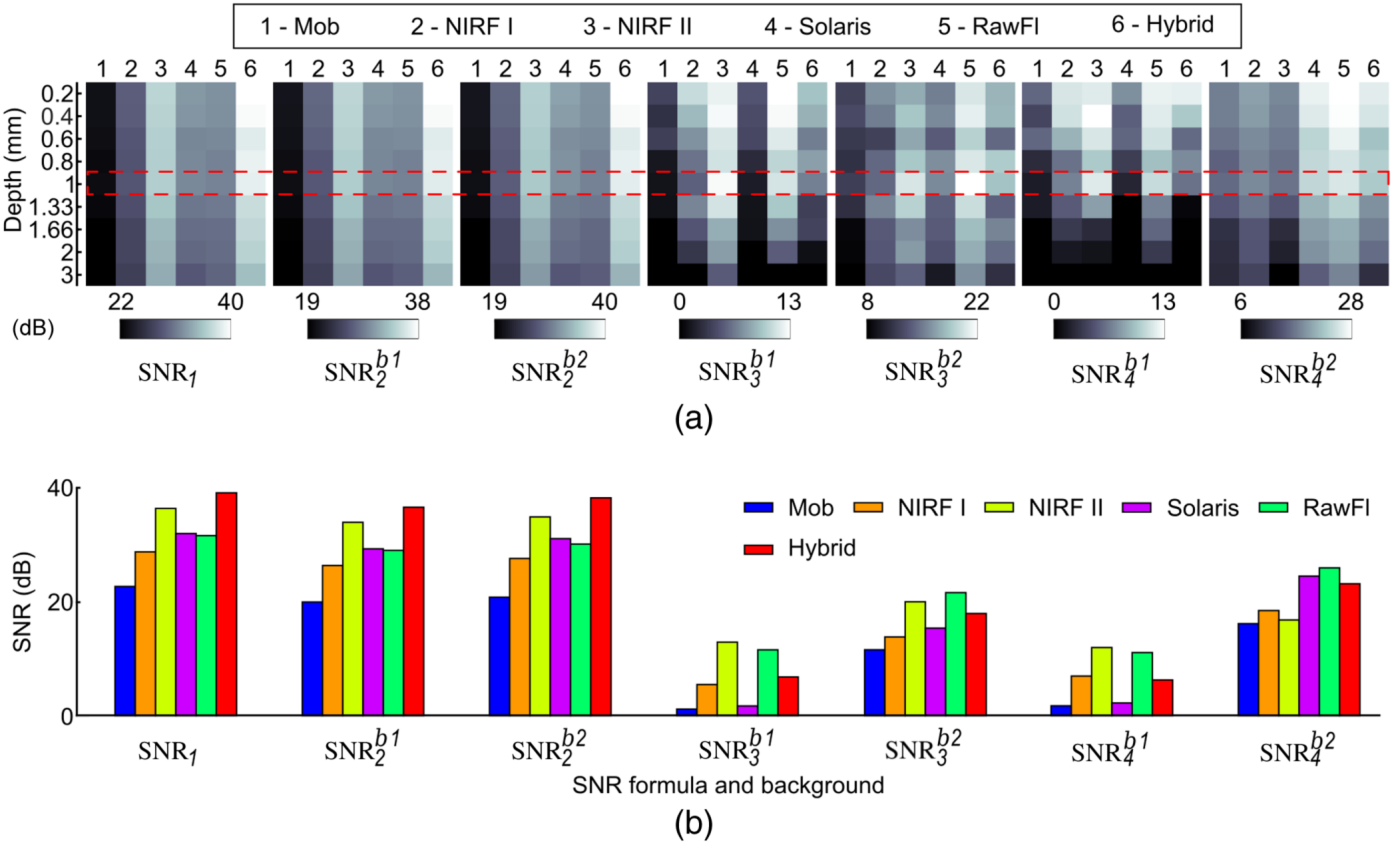
Dependence of SNR on the two background locations shown in Fig. 2(a) and/or the quantification formulas of Table 2 for different FMI systems. (a) SNR values for all systems at each depth. ${\mathrm{SNR}}_{1}$ shows the same behavior for each system as a function of depth. ${\mathrm{SNR}}_{2}$ shows a similar trend to ${\mathrm{SNR}}_{1}$ for all systems, regardless of the background employed. ${\mathrm{SNR}}_{3}$ and ${\mathrm{SNR}}_{4}$ show different trends compared with ${\mathrm{SNR}}_{1}$ and ${\mathrm{SNR}}_{2}$, depending on the background. (b) SNR values of the phantom well with depth = 1 mm for all systems. The values correspond to the dashed area highlighted in panel (a).

**Fig. 4:**
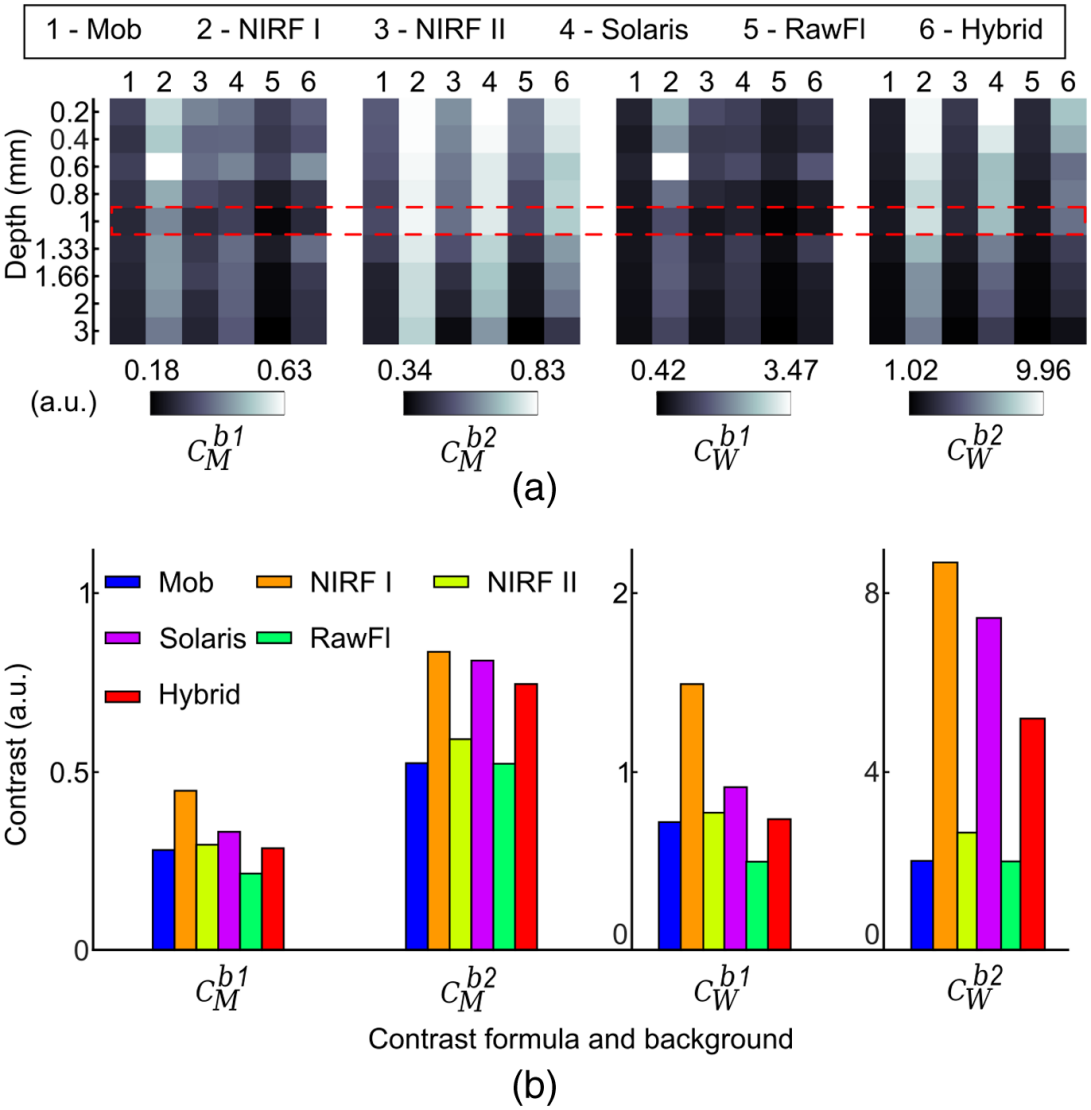
Dependence of contrast on the two background locations shown in Fig. 2(a) and/or the quantification formulas of Table 2 for different FMI systems. (a) The contrast metric results for all systems at each depth. $C_{M}$ and $C_{W}$ show similar trends when either b1 or b2 is employed for both calculations. (b) Contrast results for the phantom well with depth = 1 mm for all systems. The values correspond to the dashed area highlighted in panel (a).

Figures 3 and 4 illustrate the calculated SNR and contrast metrics, respectively, as functions of depth. The results obtained using ${\mathrm{SNR}}_{1}$ and ${\mathrm{SNR}}_{2}$ show the same trend for all systems, but not equivalent values [Fig. 3(b)]. Moreover, the results obtained using ${\mathrm{SNR}}_{3}$ and ${\mathrm{SNR}}_{4}$ not only differ from ${\mathrm{SNR}}_{1}$ and ${\mathrm{SNR}}_{2}$ but are also influenced by the chosen background area. For instance, when comparing Mob and NIRF I for ${\mathrm{SNR}}_{2}^{b1}$, it is evident from Fig. 3(b) that the NIRF I system has a greater SNR than the Mob system. However, a comparison of the NIRF I system using ${\mathrm{SNR}}_{3}^{b1}$ with the Mob system using ${\mathrm{SNR}}_{2}^{b1}$ yields the opposite conclusion.

Figure 4 demonstrates the results of the contrast metrics with respect to the applied formula (i.e., $C_{M}$—Michelson contrast and $C_{W}$—Weber contrast) and considered background ROI. The trends for both $C_{M}$ and $C_{W}$ metrics are similar for each system when the same background values are considered (i.e., b1 or b2 for both formulas). Conversely, when comparing the trends observed in $C_{M}$ under the two background values, the background influence on the quantification of the contrast metrics becomes evident [Fig. 4(b)]. For example, the Mob system has a higher contrast than the RawFl system when the Michelson contrast is applied under the b1 background for both systems. This is not true, however, when the Michelson contrast is used under b1 for the Mob system and b2 for the RawFl system. In that case, the RawFl system has a higher contrast than the Mob one [see Fig. 4(b)].

**Fig. 5:**
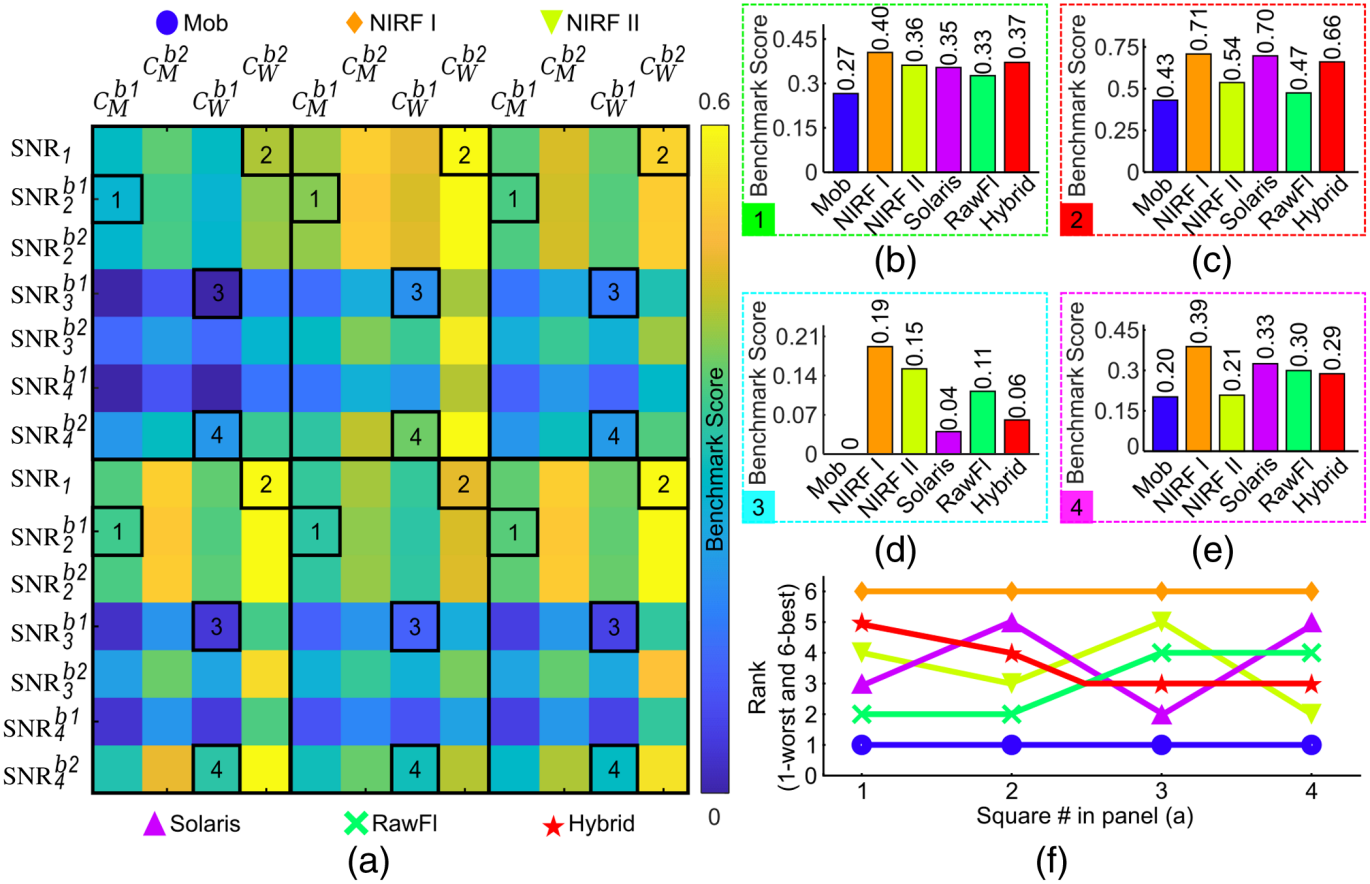
BM scores calculated according to Gorpas et al.^19^ for each system. (a) Map of the BM scores quantified using different SNRs and contrast (C) formulas (see Table 2) and two different backgrounds [see Fig. 2(a)]. The squares marked with numbers 1, 2, 3, and 4 correspond to the representative graphs of BM scores in panel (b) for square 1, ${\mathrm{SNR}}_{2}^{b1}$ and $C_{M}^{b1}$; (c) for square 2, ${\mathrm{SNR}}_{1}$; (d) for square 3, ${\mathrm{SNR}}_{3}^{b1}$; and (e) for square 4, ${\mathrm{SNR}}_{4}^{b2}$. (f) The rank of each system as a result of the BM scores for all squares of panel (a).

The influence of the applied formula and background ROI shown in Fig. 3 for SNR and Fig. 4 for contrast becomes even stronger when both metrics are combined to assess the performance of different FMI systems. Figure 5 depicts the ranking of the six systems used in this study based on the corresponding BM scores, which were calculated from the SNR and contrast metrics. Figure 5(a) illustrates the effect of combining the different formulas and background locations on the quantification of the BM scores per system. Moreover, Figs. 5(b)–5(e) demonstrate exemplary BM scores for each system as selected from the four marked squares in Fig. 5(a). The four squares were selected after a visual inspection of the map in Fig. 5(a) to showcase the variability in the quantified BM scores. As can be seen, the BM scores not only have different values, but also their trend is different per combination of formulas and background ROIs. This trend becomes clear in Fig. 5(f), where the systems’ ranking (i.e., 1—worst through 6—best) is shown for the various BM scores of Figs. 5(b)–5(e). For example, the hybrid system’s rank is superior to Solaris’ rank if their BM scores result from the combination of ${\mathrm{SNR}}_{2}^{b1}$ is used for the BM score calculation [Figs. 5(a), 5(c), and 5(f)].

## 4 Discussion and Conclusion

In the current work, through the comparison of six near-infrared FMI systems, we showed that the assessment of system performance and standardization via SNR and contrast is highly dependent on the definition of background ROI and the formulas used. This proves the need for careful attention to test a method’s clinical relevance, as well as consistency in defining metrics for objective, quantitative assessment of FMI system performance.

We used fluorescence data from the sensitivity versus depth areas of a multiparametric phantom to quantify SNR and contrast by means of different formulas obtained from the literature (Table 2). It was demonstrated that resultant SNR values can be affected by both the selected background location and the formulas applied (Fig. 3). In the case of contrast values, resultant trends appear similar for both Michelson and Weber formulas ($C_{M}$ and $C_{W}$ in Table 2), but the employed background ROI is still observed to impact the trends. Indeed, as we show in Fig. 4, the contrast ($C_{W}$) for the hybrid system changes by a factor of 8.3 depending on the background, while for the Mob system by 2.9. The dependence of the applied formula and/or background becomes more evident for SNR, where the Mob system shows a variation by a factor of 19.6 in the SNR estimation. This indicates a pressing need for common quantification formulas for SNR and contrast and consistent ROI definition for both signal and background. All measurements in this study were conducted in darkness to minimize ambient illumination that would further complicate the quantification of SNR and contrast. However, illumination is another critical factor that must be accounted for when darkness is not possible. One way to address this challenge is by acquiring a “dark” image with the excitation sources turned off and subsequently subtracting that image from the fluorescence image. This step should be performed before quantifying and reporting any performance assessment and quality control metrics. Meeting these requirements is crucial to achieving reliable results and standardization guidelines for FMI.^2^^,^^14^ Having this internal consistency during the development of FMI systems will lead to the establishment of international consensus across the field and will contribute to the widespread acceptance and use of FMI.

Our goal, however, was not only to assess the performance of each system in different SNRs and contrast definitions but also to show how these definitions affect the comparison of markedly different systems. The results of our contrast and SNR calculations were translated into BM scores and then to rank values. This analysis revealed the dependence of the ranking on the definition of background ROIs or the adopted formulas (Fig. 5). For example, the rank value for the Solaris system was lower than the corresponding values for the NIRF II and the hybrid systems if the performance assessment was based on the Michelson contrast and SNR_2_ formula with background defined as b1. However, the Solaris system ranks higher than the NIRF II and the hybrid systems when SNR is evaluated as SNR_1_ and contrast through the Weber formula with the b2. This inconsistency in the determinants of the metrics for system evaluation can affect the development and comparison of systems and ultimately the design and efficacy of clinical or pre-clinical studies. In a recent report, the American Association of Physicists in Medicine (AAPM) proposed SNR_4_ and $C_{M}$, as metrics for the performance assessment of fluorescence imaging systems.^17^ Moreover, the suggested background region proposed in these guidelines for the estimation of SNR corresponds to a region with the same optical properties as the interrogated wells, but without fluorescent dye. This corresponds to the ROI b_2_ in our study since the wells are gradually covered with the phantom matrix material which has no fluorescent dye. On the other hand, in the AAPM study, the contrast is associated with the resolution of a system and not the signal contrast as employed herein. Thus, although there is agreement in the SNR definition (${\mathrm{SNR}}_{4}^{b1}$) between the AAPM and our study, we additionally employed the contrast as a means of sensitivity assessment. Nevertheless, these recommendations represent a promising initial step toward establishing a widely accepted protocol for standardizing FMI systems, thereby addressing the inconsistencies demonstrated herein.

Similar limitations for the quantification of SNR and contrast have also been reported during the use of FMI systems in pre-clinical and clinical applications. For example, LaRochelle et al.^28^ discussed the variability of the methods used for reporting the quantitative sensitivity metrics using 3D anthropomorphic phantoms with incorporated NIR fluorescent tumor parts. On the other hand, Hoogstins et al.^21^ used data from both animal and human studies with multiple fluorescence tracers to show that background noise and background selection have a significant influence on the quantification of SBR and contrast-to-background ratio. Similarly, Azargoshasb et al.^25^ showed that SBR quantification can impact the surgical discrimination of fluorescence signals, highlighting the importance of the applied quantification approach in intraoperative decision-making. Herein, we present, to the best of our knowledge, the first study that showcases not only how the adopted formulas and the used background affect the performance assessment of an FMI system but also how the lack of consensus on quantification methods of SNR and contrast can result to misleading interpretation of system comparison measurements.

Moreover, for the quantification of the BM scores, we assumed normal signal distributions, according to which a measurement represents 95% confidence when its value is twice the magnitude of the noise level.^19^ Thus, the reference threshold values applied here are user-independent in comparison to another value commonly used in fluorescence imaging, the Rose criterion.^17^ The Rose criterion method also sets a limit of detection for fluorescence imaging for which the CNR values must be greater than 3 to 5.^40^ However, the range of a particular threshold value varies from study to study^35^^,^^41^^,^^42^ and depends on several parameters such as object shape, edge sharpness, viewing distance, and observer experience. Besides the parameters affecting the threshold value, Rose’s studies were intended for electronic imaging systems (i.e., photography, television, and optical and visual systems)^43^ and were focused on human perception of signal detectability.^44^ However, threshold values that are constrained by aspects of the human visual system might no longer be relevant with the advent of artificial intelligence (AI) imaging and signal processing. AI algorithms will allow for lower thresholds according to definitions that are not subject to intra- and inter-human observer variability.^45^^–^^47^ The criterion adopted herein follows a more simplistic statistical approach that evaluates system performance without depending on human perception and thus is more relevant for assessing the detection limits of FMI systems.

The findings of this study are also relevant to existing ICG-based FIGS systems. Similar to FMI, most FIGS system sensitivity assessment and quality control approaches are still based on the quantification of SNR and contrast metrics. However, the quantification methods for these metrics still represent a major limitation factor for cross-platform system comparisons and affect the design and/or repeatability of preclinical or clinical trials. Moreover, consistency in quantification and reporting of the various performance assessment metrics is especially important for FIGS systems, as no established quality control protocols currently exist despite the wide clinical use of such systems. The quantitative assessment of the system performance presented herein advances the current standardization strategies, which is critical for the further development of this technology and for establishing the performance limits that are a prerequisite for regulatory approvals.

Finally, similar challenges in the quantification of SNR and contrast are present in other optical technologies that are currently under investigation. For example, Palma-Chavez et al.^26^ showcased variability in SNR and contrast quantification methods within the field of optoacoustics. Fluorescence lifetime imaging is another emerging and very promising technology that also lacks consensus in the quantification of SNR, despite its frequent use in assessing the reliability of lifetime measurements. Under appropriate modifications, our study can also be adapted for such technologies, thereby contributing to the development of performance assessment and quality control protocols for imaging methods beyond FMI and FIGS.

## Disclaimer

The mention of commercial products, their sources, or their use in connection with material reported herein is not to be construed as either an actual or implied endorsement of such products by the U.S. Department of Health and Human Services. This paper reflects the views of the authors and should not be construed to represent the U.S. FDA’s views or policies.

## Data Availability

The code for estimating the SNR and contrast and the data presented herein are available on GitHub (https://github.com/IBMIfluoLab/SNRandContrast4FMI).

## References

[1] Koch M. Symvoulidis P. Ntziachristos V., “Tackling standardization in fluorescence molecular imaging,” Nat. Photonics 12(9), 505–515 (2018). NPAHBY 1749-4885 10.1038/s41566-018-0221-5

[2] Pogue B. W. et al., “Fluorescence-guided surgery and intervention—an AAPM emerging technology blue paper,” Med. Phys. 45(6), 2681–2688 (2018). MPHYA6 0094-2405 10.1002/mp.12909 29633297 PMC9560243

[3] van Dam G. M. et al., “Intraoperative tumor-specific fluorescence imaging in ovarian cancer by folate receptor-alpha targeting: first in-human results,” Nat. Med. 17(10), 1315–1319 (2011).1078-8956 10.1038/nm.2472 21926976

[4] Van Keulen S. et al., “The evolution of fluorescence-guided surgery,” Mol. Imaging Biol. 25(1), 36–45 (2023). 10.1007/s11307-022-01772-8 36123445 PMC9971137

[5] Hadjipanayis C. G. Stummer W., “5-ALA and FDA approval for glioma surgery,” J. Neurooncol. 141(3), 479–486 (2019). 10.1007/s11060-019-03098-y 30644008 PMC6445645

[6] Lotan Y. et al., “Blue light flexible cystoscopy with hexaminolevulinate in non-muscle-invasive bladder cancer: review of the clinical evidence and consensus statement on optimal use in the USA—update 2018,” Nat. Rev. Urol. 16(6), 377–386 (2019). 10.1038/s41585-019-0184-4 31019310 PMC7136177

[7] Tanyi J. L. et al., “Phase 3, randomized, single-dose, open-label study to investigate the safety and efficacy of pafolacianine sodium injection (OTL38) for intraoperative imaging of folate receptor positive ovarian cancer,” J. Clin. Oncol. 39(15), 5503–5503 (2021). JCONDN 0732-183X 10.1200/JCO.2021.39.15_suppl.5503

[8] Sarkaria I. S. et al., “Pafolacianine for intraoperative molecular imaging of cancer in the lung: the ELUCIDATE trial,” J. Thorac. Cardiovasc. Surg. 166(6), E468–E478 (2023). JTCSAQ 0022-5223 10.1016/j.jtcvs.2023.02.025 37019717 PMC12507096

[9] Ochoa M. I. et al., “Assessment of open-field fluorescence guided surgery systems: implementing a standardized method for characterization and comparison,” J. Biomed. Opt. 28(9), 096007 (2023). JBOPFO 1083-3668 10.1117/1.JBO.28.9.096007 37745774 PMC10513724

[10] Sevick-Muraca E. M. et al., “Imaging of lymph flow in breast cancer patients after microdose administration of a near-infrared fluorophore: feasibility study,” Radiology 246(3), 734–741 (2008). RADLAX 0033-8419 10.1148/radiol.2463070962 18223125 PMC3166516

[11] Gorpas D. et al., “Benchmarking of fluorescence cameras through the use of a composite phantom,” J. Biomed. Opt. 22(1), 016009 (2017). JBOPFO 1083-3668 10.1117/1.JBO.22.1.016009 28301638

[r12] Heeman W. et al., “A guideline for clinicians performing clinical studies with fluorescence imaging,” J. Nucl. Med. 63(5), 640 (2022). JNMEAQ 0161-5505 10.2967/jnumed.121.262975 35145017

[r13] Zhu B. et al., “Determining the performance of fluorescence molecular imaging devices using traceable working standards with SI units of radiance,” IEEE Trans. Med. Imaging 35(3), 802–811 (2016). ITMID4 0278-0062 10.1109/TMI.2015.2496898 26552078 PMC5304482

[r14] Sterkenburg A. J. et al., “Standardization and implementation of fluorescence molecular endoscopy in the clinic,” J. Biomed. Opt. 27(7), 074704 (2022). JBOPFO 1083-3668 10.1117/1.JBO.27.7.074704 35170264 PMC8847121

[r15] Koller M. et al., “Implementation and benchmarking of a novel analytical framework to clinically evaluate tumor-specific fluorescent tracers,” Nat. Commun. 9(1), 3739 (2018). NCAOBW 2041-1723 10.1038/s41467-018-05727-y 30228269 PMC6143516

[r16] Hacker L. et al., “Criteria for the design of tissue-mimicking phantoms for the standardization of biophotonic instrumentation,” Nat. Biomed. Eng. 6(5), 541–558 (2022). 10.1038/s41551-022-00890-6 35624150

[r17] Pogue B. W. et al., “AAPM task group report 311: guidance for performance evaluation of fluorescence-guided surgery systems,” Med. Phys. 51, 740–771 (2023). 10.1002/mp.16849 38054538

[r18] Anastasopoulou M. et al., “Comprehensive phantom for interventional fluorescence molecular imaging,” J. Biomed. Opt. 21(9), 091309 (2016). JBOPFO 1083-3668 10.1117/1.JBO.21.9.091309 27304578

[19] Gorpas D. et al., “Multi-parametric standardization of fluorescence imaging systems based on a composite phantom,” IEEE Trans. Biomed. Eng. 67(1), 185–192 (2020). IEBEAX 0018-9294 10.1109/TBME.2019.2910733 30990172

[20] Zhu B. Rasmussen J. C. Sevick-Muraca E. M., “A matter of collection and detection for intraoperative and noninvasive near-infrared fluorescence molecular imaging: to see or not to see?,” Med. Phys. 41(2), 022105 (2014). MPHYA6 0094-2405 10.1118/1.4862514 24506637 PMC3987664

[21] Hoogstins C. et al., “Setting standards for reporting and quantification in fluorescence-guided surgery,” Mol. Imaging Biol. 21(1), 11–18 (2019). 10.1007/s11307-018-1220-0 29845427

[r22] Chen T. W. et al., “In situ background estimation in quantitative fluorescence imaging,” Biophys. J. 90(7), 2534–2547 (2006). BIOJAU 0006-3495 10.1529/biophysj.105.070854 16387783 PMC1403198

[r23] Widen J. C. et al., “Methods for analysis of near-infrared (NIR) quenched-fluorescent contrast agents in mouse models of cancer,” Methods Enzymol. 639, 141–166 (2020). MENZAU 0076-6879 10.1016/bs.mie.2020.04.012 32475399

[24] Dijkhuis T. H. et al., “Semi-automatic standardized analysis method to objectively evaluate near-infrared fluorescent dyes in image-guided surgery,” J. Biomed. Opt. 30(2), 026001 (2024). JBOPFO 1083-3668 10.1117/1.JBO.29.2.026001 PMC10833575 38312853

[25] Azargoshasb S. et al., “Quantifying the impact of signal-to-background ratios on surgical discrimination of fluorescent lesions,” Mol. Imaging Biol. 25(1), 180–189 (2023). 10.1007/s11307-022-01736-y 35711014 PMC9971139

[26] Palma-Chavez J. et al., “Review of consensus test methods in medical imaging and current practices in photoacoustic image quality assessment,” J. Biomed. Opt. 26(9), 090901 (2021). JBOPFO 1083-3668 10.1117/1.JBO.26.9.090901 34510850 PMC8434148

[27] Zhu B. Sevick-Muraca E. M., “A review of performance of near-infrared fluorescence imaging devices used in clinical studies,” Br. J. Radiol. 88(1045), 20140547 (2015). BJRAAP 0007-1285 10.1259/bjr.20140547 25410320 PMC4277384

[28] LaRochelle E. P. M. et al., “3D-printed tumor phantoms for assessment of in vivo fluorescence imaging analysis methods,” Mol. Imaging Biol. 25(1), 212–220 (2023). 10.1007/s11307-022-01783-5 36307633 PMC9970939

[29] Mahesh M., “The essential physics of medical imaging, Third Edition,” Med. Phys. 40(7), 077301 (2013). MPHYA6 0094-2405 10.1118/1.4811156 28524933

[r30] Leake M. C., “Analytical tools for single-molecule fluorescence imaging *in cellulo*,” Phys. Chem. Chem. Phys. 16(25), 12635–12647 (2014). PPCPFQ 1463-9076 10.1039/C4CP00219A 24626744

[r31] Ferrand A. et al., “Using the NoiSee workflow to measure signal-to-noise ratios of confocal microscopes,” Sci. Rep. 9(1), 1165 (2019). SRCEC3 2045-2322 10.1038/s41598-018-37781-3 30718583 PMC6361975

[r32] Michelson A. A., Studies in optics, Dover Publications, New York (1995).

[33] Peli E., “Contrast in complex images,” J. Opt. Soc. Am. A 7(10), 2032–2040 (1990). JOAOD6 0740-3232 10.1364/JOSAA.7.002032 2231113

[34] Ghassemi P. et al., “Evaluation of mobile phone performance for near-infrared fluorescence imaging,” IEEE Trans. Biomed. Eng. 64(7), 1650–1653 (2017). IEBEAX 0018-9294 10.1109/TBME.2016.2601014 28113231 PMC5360525

[35] Kanniyappan U. et al., “Performance test methods for near-infrared fluorescence imaging,” Med. Phys. 47(8), 3389–3401 (2020). MPHYA6 0094-2405 10.1002/mp.14189 32304583 PMC7496362

[36] Ségaud S. et al., “Trident: a dual oxygenation and fluorescence imaging platform for real-time and quantitative surgical guidance,” Front. Photonics 3, 1–12 (2022). 10.3389/fphot.2022.1032776

[37] Glatz J. et al., “Concurrent video-rate color and near-infrared fluorescence laparoscopy,” J. Biomed. Opt. 18(10), 101302 (2013). JBOPFO 1083-3668 10.1117/1.JBO.18.10.101302 23797876

[38] Behrooz A. et al., “Multispectral open-air intraoperative fluorescence imaging,” Opt. Lett. 42(15), 2964–2967 (2017). OPLEDP 0146-9592 10.1364/OL.42.002964 28957220

[39] Tenditnaya A. et al., “Performance assessment and quality control of fluorescence molecular endoscopy with a multi-parametric rigid standard,” IEEE Trans. Med. Imaging (2024). ITMID4 0278-0062 10.1109/TMI.2024.3398816 38717879

[40] Cherry S. R. et al., Physics in nuclear medicine, 4th ed., Elsevier/Saunders, Philadelphia (2012).

[41] Davis S. C. et al., “Contrast-detail analysis characterizing diffuse optical fluorescence tomography image reconstruction,” J. Biomed. Opt. 10(5), 050501 (2005). JBOPFO 1083-3668 10.1117/1.2114727 16292936

[42] Won N. et al., “Imaging depths of near-infrared quantum dots in first and second optical windows,” Mol. Imaging 11(4), 338–352 (2012). 10.2310/7290.2011.00057 22954148

[43] Hsieh S. S. et al., “A minimum SNR criterion for computed tomography object detection in the projection domain,” Med. Phys. 49(8), 4988–4998 (2022). MPHYA6 0094-2405 10.1002/mp.15832 35754205 PMC9446706

[44] Burgess A. E., “The Rose model, revisited,” J. Opt. Soc. Am. A Opt. Image Sci. Vision 16(3), 633–646 (1999). 10.1364/JOSAA.16.000633 10069050

[45] Hossain M. S. et al., “Region of interest (ROI) selection using vision transformer for automatic analysis using whole slide images,” Sci. Rep. 13(1), 11314 (2023). SRCEC3 2045-2322 10.1038/s41598-023-38109-6 37443188 PMC10344922

[r46] Veiga-Canuto D. et al., “Comparative multicentric evaluation of inter-observer variability in manual and automatic segmentation of neuroblastic tumors in magnetic resonance images,” Cancers 14(15), 3648 (2022). 10.3390/cancers14153648 35954314 PMC9367307

[47] Tizhoosh H. R. et al., “Searching images for consensus can AI remove observer variability in pathology?,” Am. J. Pathol. 191(10), 1702–1708 (2021). AJPAA4 0002-9440 10.1016/j.ajpath.2021.01.015 33636179

